# Efficient Image Super-Resolution via Self-Calibrated Feature Fuse

**DOI:** 10.3390/s22010329

**Published:** 2022-01-02

**Authors:** Congming Tan, Shuli Cheng, Liejun Wang

**Affiliations:** 1 College of Information Science and Engineering, Xinjiang University, Urumqi 830046, China; smartan1997@stu.xju.edu.cn (C.T.); wljxju@xju.edu.cn (L.W.); 2 College of Mathematics and System Science, Xinjiang University, Urumqi 830046, China

**Keywords:** super-resolution, lightweight networks, reconstruction effect

## Abstract

Recently, many super-resolution reconstruction (SR) feedforward networks based on deep learning have been proposed. These networks enable the reconstructed images to achieve convincing results. However, due to a large amount of computation and parameters, SR technology is greatly limited in devices with limited computing power. To trade-off the network performance and network parameters. In this paper, we propose the efficient image super-resolution network via Self-Calibrated Feature Fuse, named SCFFN, by constructing the self-calibrated feature fuse block (SCFFB). Specifically, to recover the high-frequency detail information of the image as much as possible, we propose SCFFB by self-transformation and self-fusion of features. In addition, to accelerate the network training while reducing the computational complexity of the network, we employ an attention mechanism to elaborate the reconstruction part of the network, called U-SCA. Compared with the existing transposed convolution, it can greatly reduce the computation burden of the network without reducing the reconstruction effect. We have conducted full quantitative and qualitative experiments on public datasets, and the experimental results show that the network achieves comparable performance to other networks, while we only need fewer parameters and computational resources.

## 1. Introduction

The essence of the SR task is to reconstruct the high-resolution (HR) image from a low-resolution (LR) image. The SR task is a hot and challenging point in low-level computer vision, which is mainly based on the fact that the reconstruction of a LR image into a HR image has different reconstruction directions with different environments, so it is inherently ill-posed. The problem is essentially a one-to-many relationship, which is difficult to solve with a specific mathematical relation formula. To solve this problem, many traditional methods have been proposed (e.g., interpolation-based methods [1] and degenerate model-based methods [2]), but their reconstruction results are not satisfactory. With the booming rise of deep learning (DL) techniques, convolutional neural networks (CNNs) have been attempted to constrain the solution space from LR to HR and have shown excellent performance.

Dong et al. [3] first applied CNN to the SR task and constructed the SRCNN model by establishing a direct relationship between LR and HR, which is obviously superior to the traditional non-DL method. Then, inspired by traditional sparse coding, Wang et al. [4] proposed a progressive upsampling method to achieve better HR generation at large upsampling factors (e.g., ×4). Due to the outstanding performance of the VGG [5] network on the ImageNet classification task, Kim et al. [6,7] increased the depth of the SR network to 20 layers to achieve better performance and showed that it greatly outperformed the SRCNN. We found that a deeper network model had a significant impact on improving the reconstruction performance of the network. But deeper networks are not conducive to their training, so some researchers have introduced higher learning rate and residual learning to alleviate this problem. Meanwhile, in order to effectively reduce parameters in the network, recursive learning was introduced in the DRCN [7] proposed by Kim et al. to save network parameters significantly. Similarly, DRRN [8] proposed by Tai et al. not only uses recursive learning to save network parameters but also introduces global and local residuals to promote the network training. Meanwhile, the MemNet [9] proposed by Tai et al. solves the CNN long-term dependency problem by applying recursive units and gate units. However, both algorithms require a long time and substantial graphical memory consumption during the training and testing phases. The main reason is that the first stage of these two models is to upsample LR, and the whole network process is to train the upsampled images without any downsampling operation, which introduces great computational consumption.

**Figure 1:**
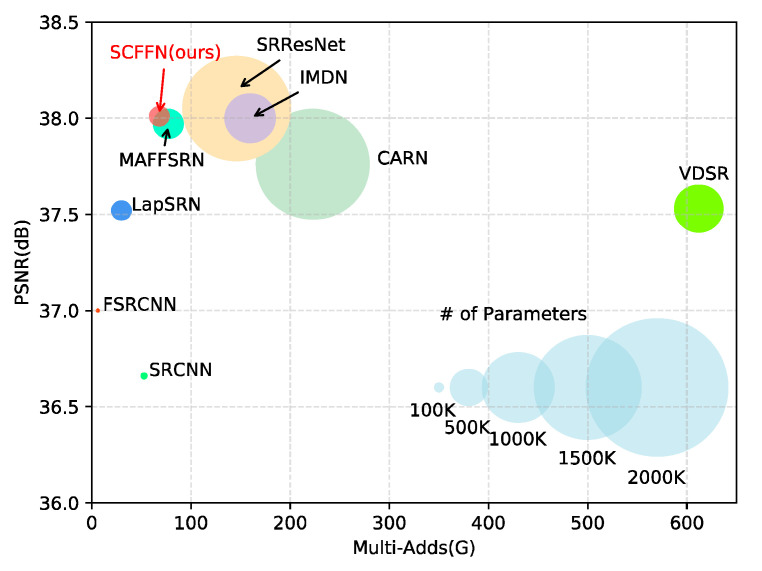
Performance comparison of our algorithm with other lightweight algorithms on Set5 (×2), where the horizontal coordinates represent the computational complexity of the network (Multi-Adds), the vertical coordinates represent the PSNR of the network and the size of the circles in the figure indicates the network parameters. Multi-Adds are computed on 720p HR images.

To address the above issues, we further explore the lightweight and reconstruction performance of single image SR network. In this paper, we propose a simple and efficient SR network via Self-Calibrated Feature Fuse (SCFFN) to achieve better balance between network performance and applicability. In the network proposed in this paper, the two crucial parts of the proposed network are the high-level semantic information learning part and the reconstruction part. In the deep feature extraction part, we propose the SCFFB according to the recent self-calibrated convolution [10]. The SCFFB has no complicated connection structure and up/down sampling operation as these are unfriendly to hardware acceleration. We summarize some previous work and find that other work is to use transpose convolution and sub-pixel convolution layer to implement image upsampling, and there is little work to study the impact of the reconstruction component on network performance and efficiency, but this structure is less efficient. In the network proposed in this paper, we use traditional NN and convolutional layers to improve the efficiency of the network and reduce parameters, while our introduced SCA improves the network performance at a small parameters cost. Therefore, the reconstruction part contains NN, SCA and convolutional layers. In general, because our network has no complicated connection mode, SCFFN is simpler and more efficient than the previous methods. As can be seen in Figure 1, our network achieves relatively optimal results in terms of parameters, model complexity and network reconstruction performance compared to the previous network.

The main contributions of this work are as follows:

- We have carefully designed a simple and effective lightweight SR network (SCFFN), and extensive experimental results demonstrate the superiority of our network over other networks.
- Inspired by self-calibrated convolution [10], we constructed a novel high-level feature learning block (SCFFB) for the SR task, which enables extract high-level information by its own feature fusion.
- To further improve the efficiency of the network, we used the traditional nearest neighbor interpolation method and the simple channel attention mechanism we designed in the reconstruction part, named U-SCA block.

## 2. Related Work

In recent years, supervised deep learning methods have been introduced to various computer vision tasks [11] and have achieved remarkable success. Garcia et al. [12] summarized the application and achievements of deep learning in semantic segmentation in recent years. At the same time, to compensate for the disadvantage that supervised learning requires a large number of labels, researchers have proposed a semi-supervised learning method, where there is only a small amount of label data and a large amount of unlabel data. The mean teachers method proposed by Vainen et al. [13] effectively improves the network performance of semi-supervised learning by averaging model weights. Doulamis et al. [14] proposed a semi-supervised learning method for object classification/tracking, which allowed the unsupervised data to initially configure the network, and then triggered the gradient descent optimization scheme to fine-tune the data. In addition, an adaptive method was proposed, which allowed the model to be dynamically modified according to the current visual conditions. Baur et al. [15] embedded the concept of auxiliary manifold of semi-supervised learning into FCNs to solve the segmentation of MS lesions. For SR tasks, numerous CNN-based methods have been proposed [16,17,18,19,20] to improve the reconstruction performance of the network, however, the network parameters and computational consumption limit their application in industry [21].

Dong et al. [3] constructed the first super-resolution reconstruction method based on deep learning through three convolution layers, named SRCNN, whose reconstruction performance is obviously better than that of traditional methods, but the input of SRCNN is bicubic interpolated image, which greatly increases the computational cost and training time. To improve the reconstruction performance, the VDSR proposed by Kim et al. [6] used global residual learning to expand the network to 20 layers, and their experimental results demonstrated that increasing the depth of the network could improve the performance of the network. Later, more and more researchers designed very deep and complex networks to improve network performance, but the consequence was that the network parameters, complexity and training cost were greatly increased. For example, EDSR [19] achieved an unprecedented breakthrough in image reconstruction performance and won the 2017 NTIRE competition, but the network parameters and depth were 43 M and 69 layers respectively. Zhang et al. proposed RDN [16] by introducing dense connection in the residual block of EDSR, which includes 22 M parameters and 128 layers. Meanwhile, Zhang et al. proposed RCAN [17] by applying the channel attention mechanism to the residual structure for the first time, which greatly improved the network performance. The network depth reached 400 layers but only needed 15.59 M network parameters. Although these methods achieve good performance, they are not suitable for use in devices with limited resources. For mobile devices, in the case of limited available memory and inference time, we should aim at the highest possible SR performance. Moreover, many situations (such as video applications, smartphones, edge devices, etc.) require good performance and faster reconstruction speeds. It is therefore essential to design a lightweight and efficient model to meet these requirements. However, most of the algorithms mentioned above have a large number of parameters and require much more memory consumption, so these networks are unaffordable for mobile devices with limited resources in practical applications.

Many fast, lightweight networks have been designed to address these issues. To accelerate network training and reduce computational costs, FSRCNN [22] implements network upsampling in the final stage of the network. This structure enables the whole network to learn high-level information in the low-dimensional space. Meanwhile, the ESPCN [23] proposes an effective sub-pixel convolutional layer to implement image upsampling. In order to reduce network parameters, some researchers adopt parameter sharing and recursive structure. For example, the DRCN [7] proposed by Kim et al. is the first to apply recursive structures to the SR task, while introducing residual connection to alleviate the gradient disappearance caused by too many recurrences. The DRRN [8] proposed by Tai et al. introduced the recursive structure deeper through gradient cropping and achieved good results. Meanwhile, Tai et al. adopted recursive units and gate units to solve the long-term dependency problem of the network and proposed MemNet [9] for multi-tasks (including image denoising, SR and JPEG deblocking). The abovementioned methods all adopt recursive structure and parameter sharing to reduce the complexity of the model. Ahuja et al. introduced the Laplace pyramid structure to the SR task to address the speed and reconstruction performance of SR, proposing LapSRN [24] and MS-LapSRN [25], both of which use LR images as input to progressively reconstruct multi-scale HR images. Similar to this work, the ProSR [26] proposed by Perazzi et al. took this structure while reconstructing higher quality images at large scaling factors. The CARN [16] proposed by Ahn et al. uses cascade connection to efficiently transmit information, thus realizing the lightweight of the network. SRResNet [27] improves the performance by removing unnecessary blocks. Later, Hui et al. proposed lightweight IDN [18] and IMDN [28] based on information distillation. In a word, it is of great significance to design a lightweight and efficient SR network.

In this paper, we further explored the lightweight and reconstruction performance of the SR network. Inspired by self-calibration convolution [10], we designed a simple and efficient SR network, namely SCFFN. Consistent with the learning-based reconstruction method (e.g., pixel shuffling [29]) that realize upsampling at the last stage of the network. However, the reconstruction module of most networks consists of upsampling (sub-pixel convolution or transpose convolution) and convolution layers. The reconstruction part in SCFFN adopts NN, the SCA of our design and two convolutional layers. We conclude from previous work that attention mechanisms [30,31] can improve network performance, but there is little work on the impact of the reconstruction stage on network performance. Therefore, in this work, we employ an attention mechanism-based U-SCA block in the reconstruction phase to better reconstruct images. Sufficient experiments have shown that our network is superior to most methods in parameters and complexity.

## 3. Method

In this part, the proposed network structure is described in detail. Section 3.1 introduces the overall framework and implementation process of our network. Section 3.2 describes in detail SCFFB, which is the core of our algorithm. Section 3.3 shows the reconstruction module of our network. Section 3.4 presents the loss function we need to train the network.

### 3.1. Network Architecture

**Figure 2:**
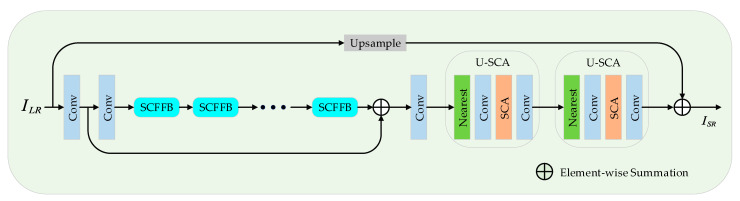
The proposed network structure of SCFFN.

Figure 2 shows the general architecture of SCFFN, which consists of three modules, the feature extraction block (FEB), the high-level semantic information learning module (i.e., a stacked series of SCFFB) and the reconstruction module (U-SCA).

The LR image is first fed to the FEB for shallow feature extraction, which consists of two convolution layers. The FEB can be expressed as:

(1) $$f_{0\;}=Conv\left(I_{LR}\right)$$

(2) $$f_{1}=Conv\left(f_{0}\right)$$

where Conv indicates a convolutional layer with kernel 3 for shallow feature extraction and $f_{1}$ is the extracted feature.

We then use a series of stacked SCFFB as the nonlinear mapping module for the network in this work to generate a powerful representation of the LR image high-level features $f_{n}$. We denote the SCFFB as $H_{SCFFB}\left(\cdot \right)$, the shallow features $f_{1}$ flow through the nonlinear feature mapping module to obtain the high-level features $f_{n}$, which can be expressed as:

(3) $$f_{n}=H_{SCFFB}^{n}\left(H_{SCFFB}^{n-1}\left(\ldots H_{SCFFB}^{1}\left(f_{1}\right)\;\right)\;\right)$$

where $f_{n}$ is the output feature map of the *n*-th SCFFB.

The skip connection is used to supplement the details of the original information to the obtained $f_{n}$, which can also effectively prevent the gradient from vanishing:

(4) $$F_{n}=f_{n}+f_{0}$$

where, + is element-wise addition, $F_{n}$ is the final feature map of low resolution.

Finally, the NN, the SCA we designed and two convolutional layers as the reconstructed part of SCFFN. We first upsample the feature map $F_{n}$ to the target size through NN and then performed feature enhancement by modeling the feature map channels (SCA). In addition, we employ a global skip connection $f_{UP}$, and the high-level semantic information learned by the network is summed element-wise to obtain a detail-rich super-resolution image. As a result, we obtain:

(5) $$I_{SR}=H_{UP}\left(F_{n}\right)+f_{UP}\left(I_{LR}\right)$$

where $H_{UP}\left(\cdot \right)$ is the reconstruction unit in our network, $I_{SR}$ is the high-resolution image after the final reconstruction of our network, and $f_{UP}$ indicates the perform bilinear interpolation operation.

Table 1 shows the parameter settings for our network. “Input” and “Output” denote the input and output flowing through the corresponding network layers, respectively. “Layers” represents the number of corresponding layers. SCFFB is the high-level information extraction block with the number of 12 (discussed in Setion 4 of the article), NN is the nearest neighbour interpolation upsampling method, and “s” is the scaling factor. It is worth mentioning that when s = 4, in order to reduce the serious mosaic and sawtooth phenomenon of the image caused by too large an upsampling factor, we split it into two ×2 upsampling.

### 3.2. Self-Calibrated Feature Fuse Block

As the core part of the SCFFN, the nonlinear mapping module consists of a series of stacked SCFFBs. SCFFB performs feature crossing to refine features. It first feeds the input feature map into the two branches and then strengthens the common part by element-wise product while increasing the nonlinear capability of the network. By multiple SCFFBs, the boundaries of the high-level features are sharpened. At the same time, we add a local skip connection to SCFFB, which can avoid the gradient disappearance caused by multiple products and compensate for the low-frequency information.

**Figure 3:**
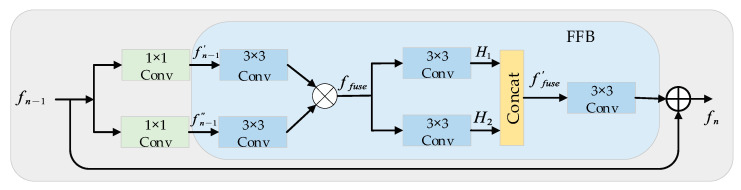
The proposed SCFFB, as the critical part of our network, for high-level semantic information extraction of LR images.

Here, we use $f_{n-1}$ and $f_{n}$ to denote the input and output of the n-th SCFFB respectively. Similar to SCNet [10], the SCFFB contains two parts. To reduce the complexity of the proposed network, we first reduce the dimension in the upper part by 1 × 1 convolution, FFB then performs feature refinement to enhance the common parts among features, producing a feature map with clear boundaries. The under part is a local residual connection to retain the original details. The SCFFB structure is shown in Figure 3. For the input feature $f_{n-1}$, we have:

(6) $$f_{n-1}^{\prime }=Conv\left(f_{n-1}\right)$$

(7) $$f_{n-1}^{\prime \prime }=Conv\left(f_{n-1}\right)$$

where $f_{n-1}^{\prime }$ and $f_{n-1}^{\prime \prime }$ are only half of the number of channels of $f_{n-1}$, Conv means that 1 × 1 convolution layer is used for dimension reduction.

The structure of FFB is shown in Figure 3. The upper part of FFB is about up-down symmetry. The feature $f_{n-1}^{\prime }$ and $f_{n-1}^{\prime \prime }$ first pass through the 3 × 3 convolution layer to adapt to the subsequent changes while increasing the nonlinear capability of the network. Then perform an element-by-element multiplication to highlight the high frequency information of the image, to achieve the transformation and fusion of the features. The fused features have clear boundaries and rich semantics. Finally, we use the shortcut to retain the original information of LR to generate the final output feature map $f_{n}$. The whole process is expressed as:

(8) $$f_{fuse}=Conv(f_{n-1}^{\prime })\;⊙\;Conv(f_{n-1}^{\prime \prime })$$

(9) $$H_{1}=Conv\left(f_{fuse}\right),\;H_{2}=Conv\left(f_{fuse}\right)$$

(10) $$f_{fuse}^{\prime }=Concat\left(\left[H_{1},H_{2}\right]\right)$$

(11) $$f_{n}=f_{n-1}+Conv\left(f_{fuse}^{\prime }\right)$$

where ⊙ means the element-wise product, $Concat\left(\left[H_{1},\;H_{2}\right]\right)$ is the concatenation operation of feature maps $H_{1}$ and $H_{2}$, $f_{fuse}$ and $f_{fuse}^{\prime }$ are the feature maps generated in the middle.

### 3.3. U-SCA Block

In the previous work, the reconstruction part of the network is often easily overlooked, because processing the up-sampled image will introduce a lot of parameters and computation. Therefore, the reconstruction module of SR network consists of an upsampling layer (sub-pixel convolution or transposed convolution) and a convolution layer. But the reconstruction part also has a significant impact on the reconstruction performance of the network.

**Figure 4:**
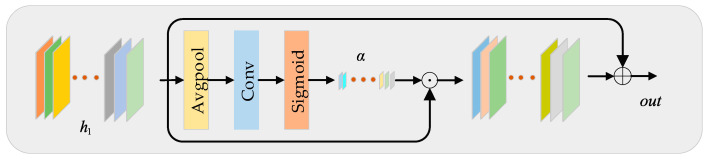
The simple channel attention (SCA) mechanism block of the network reconstruction part.

In the reconstruction stage of the network, we choose the simple and fast nearest neighbor (NN) interpolation method, which will also introduce mosaic and sawtooth effects. To alleviate this problem, this paper introduces the attention mechanism. Because convolution layer treats each channel-wise feature equally, it is unfriendly to the feature image after up-sampling by NN. For example, the network should pay attention to areas (edges, contours, etc.) with rich high-frequency information. Therefore, we modelled the interdependence among feature channels, introduce a simple channel attention mechanism (SCA) in the up-sampling phase of the network (the structure is as follows). We expect the learning of high-level features to be enhanced by explicitly modelling channel interdependencies, so that the network is able to alleviate the mosaic and jagged introduced by NN. The network structure of SCA is shown in Figure 4. The experimental results show that the SCA we introduce has a positive effect on the performance of the network, while only a few parameters need to be introduced.

In U-SCA, we first upsample the fine feature map of the nonlinear mapping learning unit to the target size by traditional NN. In order to make the network more effective and have fewer parameters, we then reduce the dimension of the upsampled feature map (i.e., reduce the number of channels) and input it into SCA for information enhancement. Finally, the number of channels is reduced three channels (i.e., RGB) by a convolution layer. The mathematical expression is:

(12) $$h_{1}=Conv(H_{NN}(f_{n}))$$

where $H_{NN\;}\left(\cdot \right)$ represents the nearest neighbour interpolation function, Conv is a 3 × 3 convolutional layer, while reducing the image dimension from 40 to 24 to ensure the efficiency of the network. $h_{1}$ is the feature map after upsampling and dimensionality reduction.

The obtained feature map $h_{1}$ is sent to SCA block to enhance the features. Specifically, firstly, through a global average pooling, then through an attention activation (Sigmoid) function layer, we get an attention weight vector $\alpha \in R^{1\times 1\times C}$, where C denotes the number of channels of the weight vector, here C=24, and finally apply the weight vector to the feature map by element-wise multiplication and addition to generate a residual map with abundant details. Its expression formula is given by:

(13) $$\alpha =Sigmiod\left(Conv\left(Avg(h_{1}\right)\right))$$

(14) $$out=\alpha \;⨀h_{1}+h_{1}$$

where Avg(·) is the global average pooling function, Sigmiod(·) is the Sigmoid function, and ⨀ is the element-wise product operation between two feature maps.

### 3.4. Loss Functions

The loss function is one of the most important parts of deep neural network, which determines the direction of our network optimization. In the SR task, because L1 [32] loss function punishes the relative error of abnormal samples less than the MSE (L2) loss function. Numerous experiments prove that the MSE loss function can produce blurred images, so in our network, the L1 loss function is used to optimize our network. The network loss function formula can be expressed as:

(15) $$L\left(\theta \right)=\frac{1}{N}\overset{N}{\underset{i=1}{\sum }}||H_{SCFFN}\left(I_{LR}^{i}\right)-I_{HR}^{i}|{|}_{1}$$

where $H_{SCFFN}\left(\cdot \right)$ denotes the network function the proposed in the paper, θ is a learnable parameter in SCFFN network, and $||\cdot |{|}_{1}$ is the $l_{1}$ norm. $\left\{I_{LR}^{i},I_{HR}^{i}\right\}$ is the training dataset pair, $I_{LR}^{i}$ and $I_{HR}^{i}$ indicate the input LR images and the corresponding ground-truth images respectively, and N represents the batch-size of training datasets.

## 4. Experiments

In this section, we verify the effectiveness of our method through sufficient experiments and the final results show its superiority. Section 4.1 introduces the proposed network training settings (such as datasets, evaluation indicators and training settings). Section 4.2 analyses each part of our network in detail, such as the impact of the number of SCFFB on performance, the effectiveness of the U-SCA, etc. Section 4.3 compares the proposed method with other algorithms in terms of objective metrics and visualization results.

### 4.1. Settings

We are using DIV2K [33] containing 800 high quality images as the training dataset. Due to the relative simplicity of the proposed network, we did not perform any data augmentation on the training dataset, but in the image preprocessing, we cut the HR images in the training dataset into small-size images as input to enhance the data. Also, the small size of the image better allows the network to learn local information. We evaluated the SR results of five standard benchmark test datasets under the peak signal-to-noise ratio (PSNR) and structural similarity (SSIM) [29]: Set5 [34], Set14 [35], BSD100 [36], Urban100 [37] and Manga109 [38]. At the same time, our model is also evaluated in the quantitative indicators of cost calculation (Multi-Adds). For a fair comparison, the results of the experimental quantitative analysis of our network, like other networks, were evaluated on the luminance (Y) channel in YCbCr channel.

Consistent with the existing network, we downsampled the ground-truth images in MATLAB using bicubic downsampling to generate LR, where the downsampling factors include (×2, ×3, ×4), and the final training dataset pair is formed.

We set the input batchsize to 32 to train our network. Also, to ensure that our network can fully learn the information in LR, we set the patch-size of LR input to 64. Meanwhile, we use Adam [25] and MultiStepLR learning scheme to optimize the network parameters. In Adam, we set $\beta_{1}=0.9$, $\beta_{2}=0.99$, and $\epsilon =10^{-8}$, and the initial learning rate is $7\times 10^{-4}$. For every 250,000 iterations, the learning rate was cropped by 0.5, and the total of 1,000,000 iterations are trained. We use Pytorch deep learning framework to implement our algorithm and train it in TITAN RTX.

### 4.2. Model Analysis

We first explored the number of SCFFB, then verified the effectiveness of U-SCA and compared the effects of other upsampling (such as transpose convolution, etc.). Finally, we qualitatively compare and visualize the proposed methods.

#### 4.2.1. Number of SCFFB Studies

**Figure 5:**
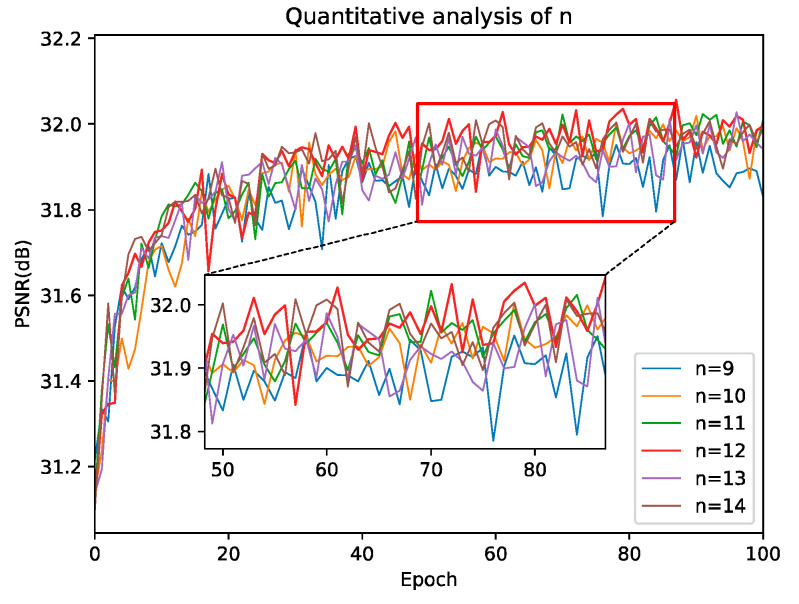
Number n of SCFFB vs. PSNR analysis, the result is evaluated on Set5 (×4).

In this section, we analyze the influence of the number of SCFFB on the performance of network through experiments. As the core component of our network, the number of SCFFB affects the final performance of our network to some extent. We should not only consider the performance of the network, but also pay attention to the parameters and computation of the network. As shown in Figure 5 and Table 2, the results show that when *n* = 12, 13, 14, the reconstruction results of the network are close. We know that the larger the n, the deeper the network, and the more network parameters and computation. The purpose of this paper is to explore the lightweight of SR network. Therefore, when the results are similar, we choose a model with relatively few parameters and calculations, so in this paper, *n* = 12 is chosen as our final network. It should be noted that SCFFN + (*n* = *i*), Parameters, Multi-Adds, PSNR and SSIM in Table 2 represent the corresponding network parameters, complexity and the average PSNR/SSIM of five common datasets on ×4 when the number of SCFFB is *i*.

#### 4.2.2. Ablation Study

**Figure 6:**
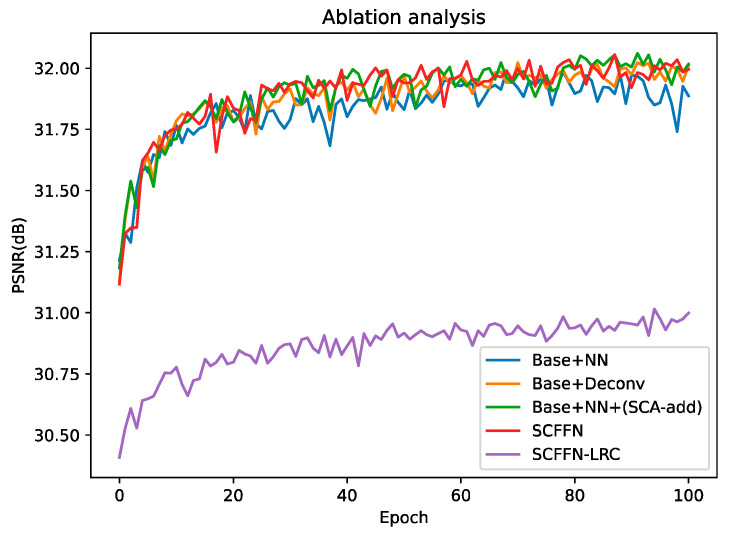
Ablation experiment analysis, the result was evaluated in Set5 (×4).

As can be seen from Figure 2 of the network architecture proposed in this paper, our network adds a local residual connection (LRC) (as seen in Equation (4)) to supplement the original rough information of LR and effectively conduct gradient transmission. We can see from Table 3 and Figure 6 that the LRC is critical to the performance improvement of our network. Due to the network is deeper, the weights of the shallow network may not be updated in time during the training process of the network, causing a significant drop in the performance of the network. Therefore, the LRC is essential in our network. SCFFN-LRC indicates removal of the LRC from the SCFFN network (see Equation (4)).

We also made a detailed experimental comparison on the reconstruction part of the network. Firstly, we replace the reconstruction part of SCFFN with NN and two convolutional layers, named Base + NN. Then, like other networks, we use transposed convolution to perform upsampling, where kernel_size = 6/7/8, padding = 2/2/2 and stride = 2/3/4 to achieve ×2/×3/×4 perceptibly, denoted Base + Deconv. At the same time, we also made an experimental comparison of U-SCA, we removed the addition branch in SCA, denoted as Base + NN + (B-add). The results of the ablation experiment are presented in Table 4 and Figure 6, where the deconvolution layer dramatically increases the parameters of the network without increasing the performance of the network, while we find that the Multi-Adds for just one transposed convolution is 94.4 G, thus demonstrating that the reconstruction part of our design improves the performance of the network at a small cost. We also find from Table 4 that the designed SCA also has a positive effect on the network performance improvement. Overall, our well-designed reconstruction part is crucial to our network. It is worth mentioning that the “Base” in Table 4 refers to the network after the reconstruction part is removed by SCFFN, the PSNR/SSIM in the results of Table 3 and Table 4 is the average value evaluated on five common test datasets (×4), the experimental results in Figure 6 are tested in Set5 (×4).

#### 4.2.3. Loss Analysis

**Figure 7:**
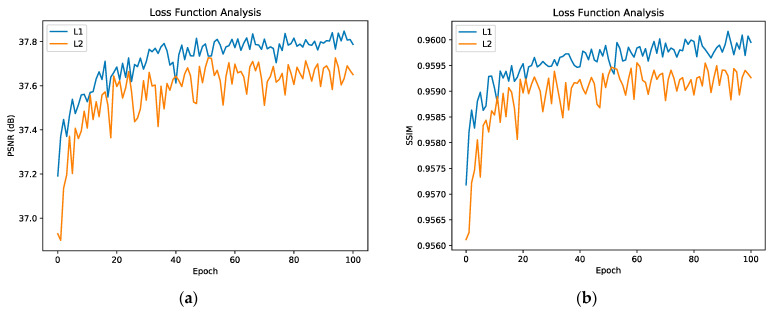
Analysis of L1 and L2 Loss Functions. (**a**) is the PSNR value evaluated on Set5 (×2), (**b**) is the SSIM value evaluated on Set5 (×2).

In this part, we explore the influence of L1 and L2 loss functions on network performance. The experimental results are shown in Figure 7, the results show that the network performance optimized by L1 loss function is better than that optimized by L2 loss function, so L1 loss function is more suitable for our network. The result is evaluated on Set5 (×2).

#### 4.2.4. Visual Analysis

**Figure 8:**
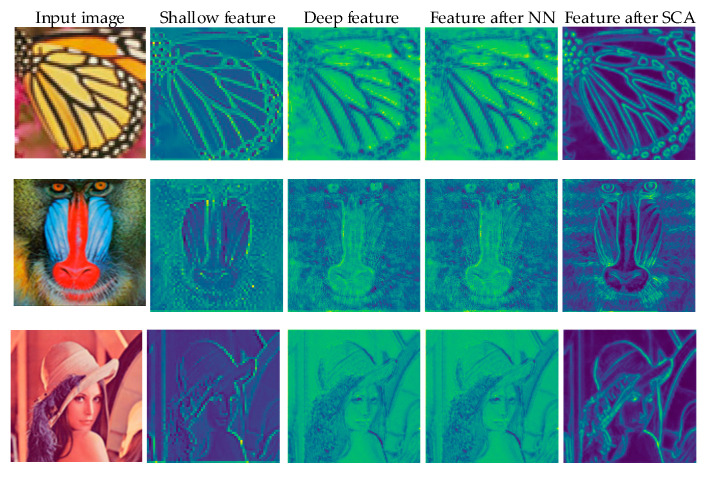
Visual feature maps.

We have visualized the intermediate feature map of the network. As shown in Figure 8. The first column represents the input image of the network, the second column represents the feature map of the image after shallow feature extraction, the third column shows the feature map after deep feature learning module, and the fourth column represents the features of NN upsampling. The last column shows the features after SCA. From these feature maps, we can find that the shallow feature map contains abundant low-frequency information. After the deep learning module, the extracted feature image retains a large amount of high-frequency information. After NN upsampling, the feature map has obvious mosaic and jaggedness phenomenon. Finally, after our proposed SCA, the high-frequency information of the image is clearly displayed, and at the same time, it can effectively alleviate the shortage of NN upsampling. Experiments show that the method we designed is very effective for lightweight SR.

### 4.3. Comparison with State-of-the-Arts

#### 4.3.1. Network Parameters

**Figure 9:**
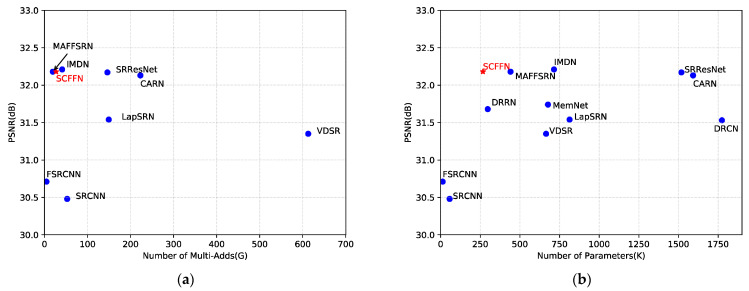
Comparison of our method with other methods in terms of Multi-Adds and Parameters. (**a**) PSNR vs. Multi-Adds. (**b**) PSNR vs. Parameters. The results shown are for experiments conducted on Set5 (×4).

After sufficient training, comparison of our model with state-of-the-art methods on the five public test datasets (see Table 5), including SRCNN [3], FSRCNN [23], VDSR [6], DRCN [7], DRRN [8], MemNet [9], CARN [21], LapSRN [24], SRResNet [27], IDMN [28], MAFFSRN [39], MADNet [40] and SMSR [41]. For a fair comparison, we only consider the models with equivalent Multi-Adds for comparison, and therefore models that were too deep and too large, such as RDN [16] and RCAN [17], were excluded here. According to the convention, we choose PSNR and SSIM [33] as metrics. The comparison results in network parameters, reconstruction effect (PSNR) and Multi-Adds (G) are shown in Figure 1 and Figure 9. Figure 1 shows that our method can balance the parameters, reconstruction performance and Multi-Adds well. It can also be seen from Figure 9a that although the Multi-Adds (27 G vs. 19.3 G) of our network is slightly higher than MAFFSRN in ×4 upscaling factors and can achieve similar performance (in Table 5 shows that we have fewer Multi-Adds on ×2). It can obtain from Figure 9b that the parameters of our network are only half that of MAFFSRN (267 K vs. 441 K). Therefore, compared with other methods, our network is lighter and more efficient. It is worth noting that Multi-Adds are estimated on 720p (1280 × 720) HR image, and Figure 1 shows our method on Set5 (×2) compared to other methods, and Figure 9 compare on Set5 (×4).

#### 4.3.2. Comparison of Reconstruction Performance and Visual Effects of the Network

In this subsection, we show the quantitative and qualitative results of SCFFN compared with state-of-the-art models (including SRCNN [3], FSRCNN [23], VDSR [6], DRCN [7], DRRN [8], MemNet [9], CARN [21], LapSRN [24], SRResNet [27], IMDN [28], MAFFSRN [39], MADNet [40] and SMSR [41]) on performance comparison on the three upscaling factors ×2, ×3 and ×4. The quantitative results of our network are presented in Table 5, which includes Multi-Adds that show the complexity of the model and parameters. Specifically, CARN has achieved comparable performance to SCFFN, but its parameters are close to 1592 K, about six times that of the proposed method. The parameters of the proposed network in this work are only 37% of IMDN, but comparable results can be achieved. Complete experimental results demonstrate that the proposed lightweight network SCFFN achieves comparable performance to other state-of-the-art methods on multiple datasets and scale factors, but we only need fewer parameters and Multi-Adds. It is worth noting that MAFFSRN is the work from the AIM 2020 Efficient SR Challenge, which ranked the network third and fourth in terms of Multi-Adds and parameters, respectively, but its code is not publicly available.

**Figure 10:**
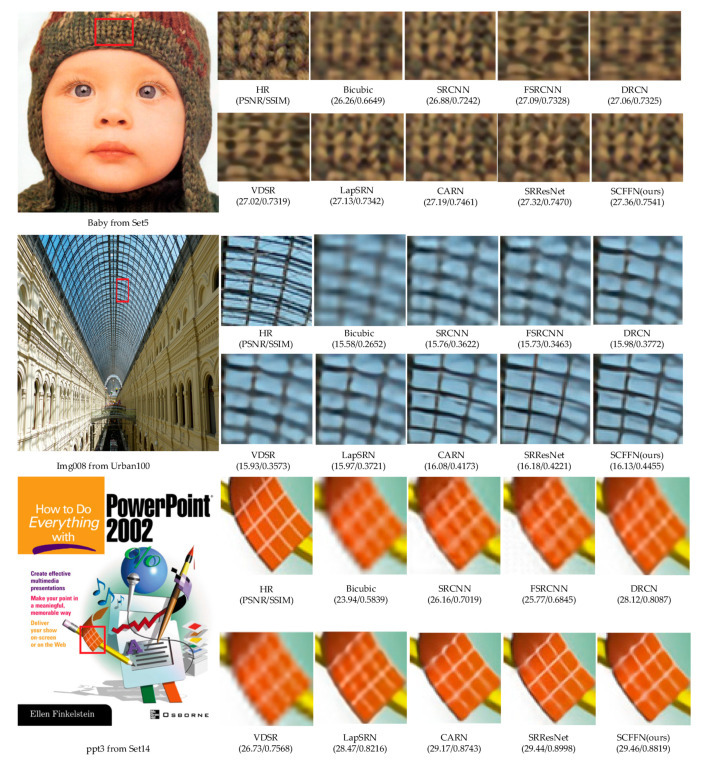
Comparison of the visual effect of the method in this paper with other methods on ×4.

We selected an image from the Set5, Set14 and Urban100 test datasets respectively for comparison of the visual reconstruction details (shown in Figure 10), we can see that our method is superior to other methods in details, such as stripes. For the image “ppt3” and “Baby”, we observe that most comparison methods will produce obvious artifacts and blurring effects, while our method produce more accurate lines. On the structural details in “img008”, the proposed network in this paper can realize reconstruction with less artifacts.

### 4.4. Discussion

Through the above ablation research and comparative experiments, we found that the image super-resolution reconstruction has great challenges in terms of trade-offs among network parameters, reconstruction performance and computational complexity, but the SR lightweight network we designed has achieved good results. However, there is room for optimization in our approach. Similar to most SR networks, it is difficult to minimize network parameters, performance and computational complexity at the same time. However, compared with other comparison networks, we only need fewer parameters and computational complexity (Multi-Adds) to achieve considerable performance.

## 5. Conclusions

In this work, we propose a lightweight network SCFFN for the SR task, in which SCFFB is the basic building block. SCFFB performs feature crossing to refine features. Specifically, the input features are first fed into two branches, and then the common part is strengthened by element-wise multiplication while increasing the nonlinear ability of the network, so that the fused features have the characteristics of clear boundary, etc. At the same time, we add the local skip connection, which not only avoids gradient dispersion caused by multiple multiplications but also supplements low-frequency information. In the reconstruction part of the network, we adopt the traditional nearest neighbor interpolation upsampling and introduce SCA to model the features channel to alleviate the mosaic and sawtooth phenomenon caused by NN. Comprehensive experiments show that the proposed method achieves comparable performance with other advanced methods, but we only need less network parameters and computational complexity.

In the future work, we will continue to explore the lightweight of SR network and try to introduce non-parametric attention mechanism or dynamic convolution layer to enhance information extraction in the high-level information learning stage of the network. In order to design a more effective up-sampling operation for the reconstruction part of the network, we can try to combine the depth separable convolution or group convolution into the transposed convolution layer to reduce the network parameters. At the same time, in the future work, we will apply this work to video SR or introduce it into the real world for real-time broadcasting.

## Figures and Tables

**Table 1:** Setting of network structure parameters of our algorithm.

Module	Layer Name	Layers	Kernel Size	Input	Output
Shallow feature extraction	Conv	1	3×3	3× 64 ×64	40× 64×64
	Conv	1	1×1	40× 64×64	40× 64×64
Deep feature extraction	SCFFB	12	1×1	40× 64×64	20× 64×64
			1×1	40× 64×64	20× 64×64
			3×3	20× 64×64	20× 64×64
			3×3	20× 64×64	20 × 64×64
			3×3	20× 64×64	20× 64×64
			3×3	20× 64×64	20 × 64×64
			3×3	40× 64×64	40× 64×64
	Conv	1	3×3	40× 64×64	40× 64×64
Network reconstruction	NN	1 or 2		40× 64×64	40× (s·64)× (s·64)
	Conv	1 or 2	3×3	40× 64×64	24× (s·64)× (s·64)
	SCA	1 or 2	3×3	24× 64×64	24× (s·64)× (s·64)
	Conv	1 or 2	3×3	24× 64×64	3× (s·64)× (s·64)

**Table 2:** Performance analysis of the number of SCFFB.

Method	Parameters	Multi-Adds	PSNR (dB)	SSIM
SCFFN + (*n* = 9)	214 K	24.2 G	28.86	0.8190
SCFFN + (*n* = 10)	232 K	25.2 G	28.87	0.8188
SCFFN + (*n* = 11)	249 K	26.2 G	28.85	0.8190
SCFFN + (*n* = 12)	267 K	27.2 G	28.93	0.8203
SCFFN + (*n* = 13)	285 K	28.2 G	28.91	0.8199
SCFFN + (*n* = 14)	302 K	29.2 G	28.92	0.8202

**Table 3:** Regarding the effect of LRC in Equation (4) on performance results, we test results on ×4 and PSNR, SSIM is the average of the test results on the five public datasets.

Method	Parameters	Multi-Adds	PSNR (dB)	SSIM
SCFFN-LRC	267 K	27.2 G	27.90	0.8023
SCFFN	267 K	27.2 G	28.93	0.8203

**Table 4:** Experimental analysis on the reconstruction part of the network. We test results on ×4 and PSNR, SSIM is the average of the test results on the five public datasets.

Method	Parameters	Multi-Adds	PSNR (dB)	SSIM
Base + NN	266 K	27.2 G	28.86	0.8188
Base + Deconv	353 K	116.8 G	28.86	0.8191
Base + NN + (SCA-add)	267 K	27.2 G	28.89	0.8194
SCFFN (ours)	267 K	27.2 G	28.93	0.8203

**Table 5:** Comparison of the results of the proposed algorithm with the state-of-the-art models on ×2, ×3, ×4.

Scale	Method	Params (K)	Multi-Adds (G)	Set5 PSNR/SSIM	Set14 PSNR/SSIM	BSD100 PSNR/SSIM	Urban100 PSNR/SSIM	Manga109 PSNR/SSIM
2	SRCNN	57	52.7	36.66/0.9542	32.45/0.9067	31.36/0.8879	29.50/0.8946	35.60/0.9663
	FSRCNN	13	6.0	37.00/0.9558	32.63/0.9088	31.53/0.8920	29.88/0.9020	36.67/0.9710
	VDSR	666	612	37.53/0.9587	33.03/0.9124	31.90/0.8960	30.76/0.9140	37.22/0.9750
	DRCN	1774	17,974	37.63/0.9588	33.04/0.9118	31.85/0.8942	30.75/0.9133	37.55/0.9732
	LapSRN	251	29.9	37.52/0.9591	32.99/0.9124	31.80/0.8952	30.41/0.9103	37.27/0.9740
	DRRN	298	6796.9	37.74/0.9591	33.23/0.9136	32.05/0.8973	31.23/0.9188	37.88/0.9749
	MemNet	678	2662.4	37.78/0.9597	33.28/0.9142	32.08/0.8978	31.31/0.9195	37.72/0.9740
	CARN	1592	222.8	37.76/0.9590	33.52/0.9166	32.09/0.8978	31.92/0.9256	38.36/0.9765
	SRResNet	1518	146.1	38.05/0.9607	33.64/0.9178	32.22/0.9002	32.23/0.9295	38.05/0.9607
	IMDN	694	159.6	37.91/0.9594	33.59/0.9169	32.15/0.8987	32.14/0.9274	38.79/0.9764
	MAFFSRN	402	77.2	37.97/0.9603	33.49/0.9170	32.14/0.8994	31.96/0.9268	-\-
	MADNet	878	178.1	37.94/0.9604	33.46/0.9167	32.10/0.8988	31.74/0.9246	-\-
	SMSR	985	131.6	38.00/0.9601	33.64/0.9179	32.17/0.8990	32.19/0.9284	38.76/0.9771
	SCFFN(ours)	256	68	38.01/0.9604	33.52/0.9169	32.12/0.8990	31.93/0.9261	38.51/0.9768
3	SRCNN	57	52.7	32.75/0.9090	29.30/0.8215	28.41/0.7863	26.24/0.7989	30.48/0.9117
	FSRCNN	13K	5.0	33.18/0.9140	29.37/0.8240	28.53/0.7910	26.43/0.8080	31.10/0.9210
	VDSR	666	612	33.66/0.9213	29.77/0.8314	28.82/0.7976	27.14/0.8279	32.01/0.9340
	DRCN	1774	17,974.3	33.82/0.9226	29.76/0.8311	28.80/0.7963	27.15/0.8276	32.24/0.9343
	DRRN	298	6796.9	34.03/0.9244	29.96/0.8349	28.95/0.8004	27.53/0.8378	32.71/0.9179
	MemNet	678	2662.4	34.09/0.9248	30.00/0.8350	28.96/0.8001	27.56/0.8376	32.51/0.9369
	CARN	1592	118.8	34.29/0.9255	30.29/0.8407	29.06/0.8034	28.06/0.8493	33.50/0.9440
	SRResNet	1554	190.2	34.41/0.9274	30.36/0.8427	29.11/0.8055	28.20/0.8535	33.54/0.9448
	IMDN	703	71.7	34.32/0.9259	30.31/0.8409	29.07/0.8036	28.15/0.8510	33.58/0.9434
	MAFFSRN	418	34.2	34.32/0.9269	30.35/0.8429	29.09/0.8052	28.13/0.8521	-\-
	MADNet	930	88.4	34.26/0.9262	30.29/0.8410	29.04/0.8033	27.91/0.8464	-\-
	SMSR	993	100.5	34.40/0.9270	30.33/0.8412	29.10/0.8050	28.25/0.8536	33.68/0.9445
	SCFFN(ours)	256	37	34.29/0.9263	30.27/0.8409	29.04/0.8034	27.98/0.8481	33.30/0.9427
4	SRCNN	57	52.7	30.48/0.8628	27.49/0.7503	26.90/0.7101	24.52/0.7221	27.66/0.8505
	FSRCNN	13	4.6	30.71/0.8657	27.59/0.7535	26.98/0.7105	24.62/0.7208	27.90/0.8517
	VDSR	665	612.6	31.35/0.8838	28.01/0.7674	27.29/0.7251	25.18/0.7524	28.83/0.8809
	DRCN	1774	17,976.3	31.53/0.8854	28.02/0.7670	27.23/0.7233	25.14/0.7510	28.98/0.8816
	LapSRN	813	149.4	31.54/0.8850	29.19/0.7720	27.32/0.7280	25.21/0.7560	29.09/0.8845
	DRRN	297	6796.9	31.68/0.8888	28.21/0.7720	27.38/0.7284	25.44/0.7638	29.46/0.8960
	MemNet	677	2662.4	31.74/0.8893	28.26/0.7723	27.40/0.7281	25.50/0.7630	29.42/0.8942
	CARN	1592	222.8	32.13/0.8937	28.60/0.7806	27.58/0.7349	26.07/0.7837	30.47/0.9084
	SRResNet	1518	146.1	32.17/0.8951	28.61/0.7823	27.59/0.7365	26.12/0.7871	30.48/0.9087
	IMDN	715	41.1	32.19/0.8936	28.57/0.7803	27.54/0.7342	26.03/0.7829	30.44/0.9065
	MAFFSRN	441	19.3	32.18/0.8948	28.58/0.7812	27.57/0.7361	26.04/0.7848	-\-
	MADNet	1002	54.1	32.11/0.8939	28.52/0.7799	27.52/0.7340	25.89/0.7782	-\-
	SMSR	1006	57.2	32.12/0.8932	28.55/0.7808	27.55/0.7351	26.11/0.7868	30.54/0.9085
	SCFFN(ours)	267	27	32.18/0.8950	28.56/0.7809	27.54/0.7352	26.01/0.7832	30.36/0.9070

## Data Availability

Publicly available datasets were analyzed in this study. Our training set DIV2k can be obtained from Available online: https://data.vision.ee.ethz.ch/cvl/DIV2K/ (accessed on 18 October 2021). The URLs of test sets Set5, Set14, BSD100, Urban 100 and Manga109 are [Low-Complexity Single-Image Super-Resolution (inria.fr)], Available online: https://sites.google.com/site/romanzeyde/research-interests (accessed on 18 October 2021), Available online: https://www.eecs.berkeley.edu/Research/Projects/CS/vision/bsds/ (accessed on 18 October 2021), Available online: https://sites.google.com/site/jbhuang0604/publications/struct_sr (accessed on 18 October 2021) and Available online: http://www.manga109.org/en/ respectively (accessed on 18 October 2021).
